# Effect of biotin on growth performance, serum biochemical profiles, and histomorphological changes in acetate gossypol-treated mice

**DOI:** 10.3389/fvets.2025.1562325

**Published:** 2025-04-15

**Authors:** Qiyu Luo, Tongxiang Xu, Zulibina Ainiwaer, Wenpeng Fan, Caidie Wang

**Affiliations:** ^1^College of Animal Sciences, Xinjiang Agricultural University, Urumqi, China; ^2^Xinjiang Key Laboratory of Herbivore Nutrition for Meat and Milk, Urumqi, China; ^3^Research Center for Biofeed and Animal Gut Health, Urumqi, China

**Keywords:** acetate gossypol, biotin, growth performance, serum biochemical profiles, histomorphological

## Abstract

The objective of this study was to determine the effects of biotin on growth performance, serum biochemical profiles, and morphology of acetate gossypol-treated mice. Seventy-five healthy male Kunming mice were randomly assigned to five treatment groups: the control group (C), the biotin control group (BC), the acetate gossypol control group (AGC), the biotin prevention group (BP) and the biotin treatment group (BT). The study examined the growth performance, visceral index, serum biochemical profiles and histomorphological changes of mice. The results showed that compared with AGC group mice, the average daily feed intake of BP group and BT group increased by 40.47 and 45.69% (*p <* 0.05). Biotin prevention reduced the elevated blood urea nitrogen (BUN) levels in acetate gossypol-treated mice, biotin treatment reduced the elevated serum aspartate aminotransferase (AST), total protein (TP) and BUN levels in acetate gossypol-treated mice (*p <* 0.05). Biotin increase the values of villus height/crypt depth in duodenum and jejunum (*p <* 0.05). In conclusion, biotin potentially increase the growth performance of acetate gossypol-treated mice, it also has a positive effect on serum biochemical profiles, and histomorphological of acetate gossypol-treated mice.

## 1 Introduction

Cotton by-products such as cottonseed meal and cottonseed protein can be used as animal feed and are important protein supplements. However, cotton by-products contain anti-nutritional factors such as gossypol. Gossypol and it’s derivatives accumulate in animal bodies and produce toxicity, which is not conducive to the growth and development of animals. Gossypol is widely distributed in the body with the blood circulation it damages the liver, kidney and other substantive organs, stimulates and destroys the gastrointestinal mucosa of animals, damages the digestive function of animals. It was found that gossypol could react with the *ε*-amino group of lysine to block the effect of trypsin, thereby reducing the utilization rate of lysine. When the gossypol content in the diet was 183 mg/kg, the malondialdehyde content in the liver tissue of the male geese decreased, with the increase of the free gossypol content in the diet, the serum triglyceride and low density lipoprotein content of the male geese increased linearly (1), the study shows that gossypol affected the lipid metabolism of the organism. Feeding 400 mg/kg gossypol could reduce the feed intake and daily gain of 21-day-old and 42-day-old male broilers (2). Gossypol will accumulate in animal tissues, reduce animal growth performance, and cause certain damage to animal viscera and intestinal health.

Vitamins are involved in the body’s biochemical reactions, and have the functions of regulating metabolism and enhancing immunity. Biotin also known as vitamin B_7_ and vitamin H, is a water-soluble B vitamin. Biotin acts as an active carrier and a cofactor in the enzymatic reaction, by forming a covalent bond with the *ε*-amino group of the lysine residue on the protein, it binds to the enzyme to form a covalent binding coenzyme of five carboxylases in mammals. These enzymes are involved in physiological activities such as gluconeogenesis, fatty acid synthesis metabolism, amino acid catabolism and energy metabolism (3). Biotin is involved in the tricarboxylic acid cycle and fat metabolism in animals, and regulates glucose metabolism and lipid metabolism in animals. Studies have found that biotin can affect the morphology and development of intestinal mucosal epithelium, regulate intestinal flora, and improve the utilization of nutrients by animals, biotin can treat colitis induced by dextran sulfate sodium in mice, restore the colon length of mice caused by colitis, and maintain the integrity of the intestinal barrier in mice (4). Biotin regulates the production of pro-inflammatory cytokines in the immune system, affecting intestinal inflammation and intestinal permeability (5). Studies have shown that after feeding mice with biotin-deficient feed for seven weeks, the body weight of mice gradually decreased, and in the liver of mice lacking biotin, the specific activities of two biotin-dependent enzymes, pyruvate carboxylase and propionyl-CoA carboxylase, decreased by 75 and 80%, indicating that biotin deficiency has a certain effect on the immune system and function of mice (6).

Since gossypol is not stable, studies always use acetate gossypol to do the experiment. Studies have found that 5 mg/(kg·BW) gossypol can reduce the reproductive capacity of male rats, resulting in a decrease in mitochondrial ATP in rats, at the same time, 100 mg/(kg·BW) vitamin E can inhibit the decrease of mitochondrial ATP (7), as one of the vitamins, vitamin E has antioxidant effect and can reduce the toxicity of gossypol in animals. As a vitamin involved in the regulation of metabolism in the body, biotin has a certain antioxidant effect and protective ability to the animal body, we hypothesized that biotin has the potential to alleviate the damage of acetate gossypol to the animal body. However, the effects of biotin on the mice treated with acetate gossypol have not been fully characterized. To date, relatively few studies have investigated the application of biotin to alleviate and protect animals from damage caused by gossypol, this work might provide theoretical basis and value reference for the application of biotin to protect mice gavaged with acetate gossypol. Therefore, in this study, the growth performance, blood biochemical indexes, visceral and intestinal tissue morphology and function of mice were detected to explore the effect of biotin on mice gavaged with acetate gossypol.

## 2 Materials and methods

### 2.1 Animals and experimental design

All experimental procedures involving animals were approved (animal protocol number: 2024009) by the Animal Welfare and Ethics Committee of Xinjiang Agricultural University, Urumqi, Xinjiang, China. Seventy-five 4-week-old healthy male Kunming mice (BW, 19.37 ± 1.86 g) were purchased from Xinjiang Medical University, randomly assigned to five treatment groups (*n* = 15), each group was reared in a cage (540 × 395 × 200 mm) and then scheduled for a two weeks acclimation period. The mice were allowed access to food and water *ad libitum* throughout the study, and their daily food consumption was estimated by weighing the remaining food. The mice basal diet was from Jiangsu Medison Biopharmaceutical Co., Ltd., Catalog Number: MD17121. All the mice were housed in the Xinjiang Agricultural University Animal Center under controlled conditions (temperature, 20–24°C; humidity, 50–60%; 12 h light/dark cycle).

**Figure 1:**
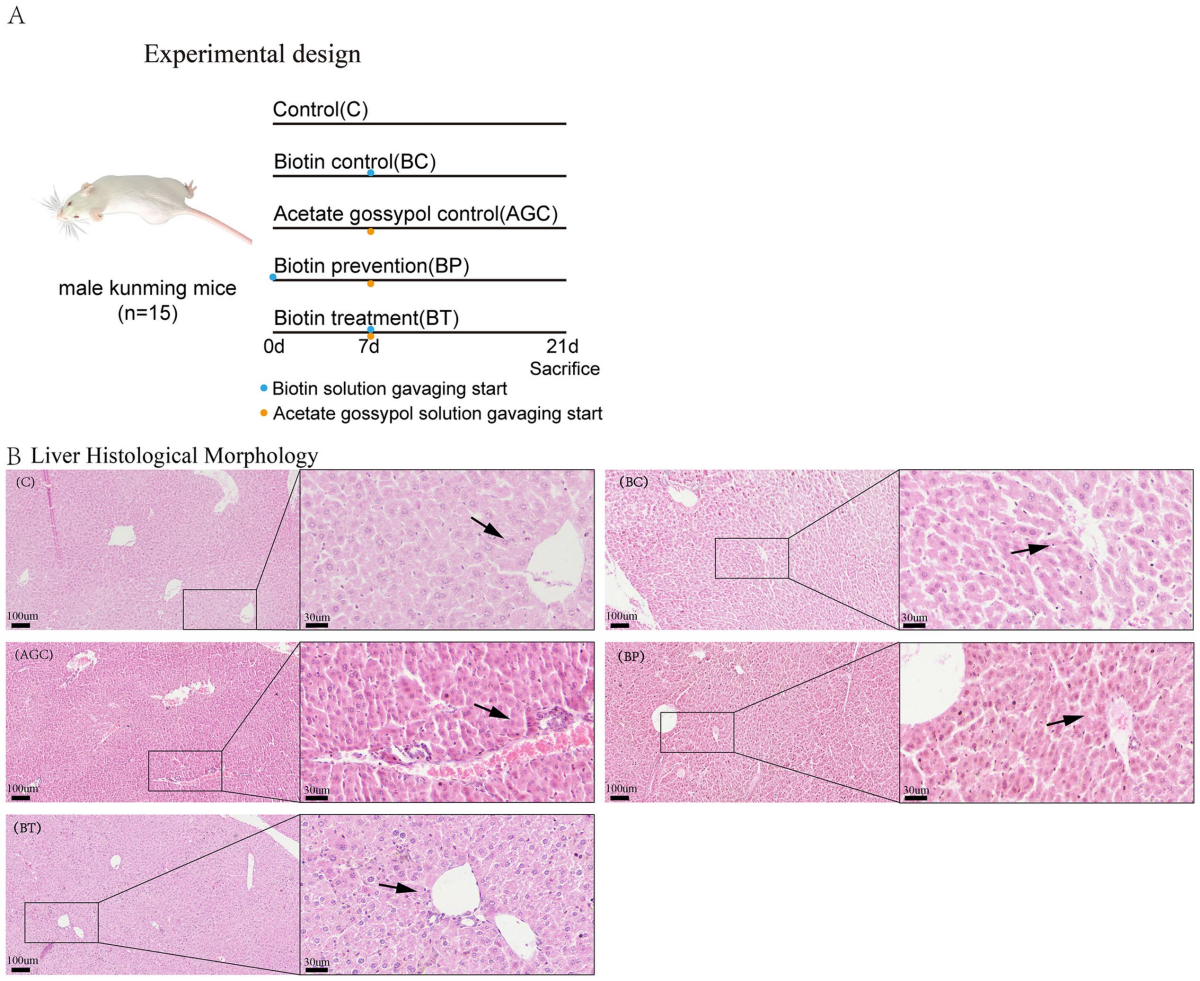
Effect of biotin on histomorphological of acetate gossypol-treated mice. **(A)** Experimental design. **(B)** Liver histological morphology (HE), magnification: 100× and 400×, the arrows indicate the liver cells surrounding the vein. C, the liver of control group mice; BC, the liver of biotin control group mice; AGC, the liver of acetate gossypol control group mice; BP, the liver of biotin prevention group mice; BT, the liver of biotin treatment group mice.

Biotin (Wuhan Servicebio Technology CO., Ltd., purity>99%) was dissolved in distilled water to make biotin solution at a dose of 1 mg/(kg·BW) of each group of mice, acetate gossypol (Medson Technology Co., Ltd., purity>98%) was dissolved in canola oil to make acetate gossypol solution at a dose of 80 mg/(kg·BW) of each group of mice. The feeding trial lasted for three weeks, biotin solution was administered to the BP group mice via oral gavage on a daily basis starting from in the first week, the remaining groups were administered equivalent volumes of distilled water and canola oil via oral gavage daily. In the second and third weeks, the control group mice were orally gavaged with distilled water and canola oil daily, the BC group mice were orally gavaged with biotin solution and canola oil daily, the AGC group mice were orally gavaged with distilled water and acetate gossypol solution daily, the BP group and the BT group mice were orally gavaged with biotin solution and acetate gossypol solution daily (Figure 1A).

### 2.2 Growth performance and visceral index

Body weight were recorded weekly and average daily feed intake of mice were recorded. Mice were euthanized by cervical dislocation, followed by immediate collection and weighing of hearts, livers, spleens, lungs, and kidneys, the visceral index was calculated according to the formula: (visceral weight/body weight) × 1,000.

### 2.3 Serum biochemistry

Blood was gained from the orbit of mice, let the blood stand at room temperature for one hour, centrifuge at 4000 r/min for 15 min, and take the supernatant as serum, store at −80°C, contents of serum urea (BUN), glucose (GLU), alanine aminotransferase (ALT), aspartate aminotransferase (AST), total bilirubin (TBIL), alkaline phosphatase (ALP), total protein (TP), albumin (ALB), triglyceride (TG), total cholesterol (TC), high density lipoprotein cholesterol (HDL-C) and low density lipoprotein cholesterol (LDL-C) were measured by Beckman au5821 Fully Automatic Biochemical Analyzer (Beckman Coulter Trading (China) Co., Ltd).

### 2.4 Histopathological observation

Following dissection of the liver, duodenum, jejunum, and ileum segments, the tissues were immediately fixed in 10% neutral buffered formalin. After fixation, specimens were systematically trimmed, dehydrated through a graded ethanol series, cleared in xylene, and embedded in paraffin blocks. Serial sections of 5 μm thickness were then cut, mounted onto glass slides and air-dried. Finally, sections were stained with hematoxylin and eosin (H&E). Slide scanning and microscopic observation were performed using the MoticEasyScan Pro 6 digital slide scanning system. Blinded histological assessment was carried out by an experienced pathologist, for each sample, three intact villi were randomly selected, and the villus height and crypt depth was recorded.

### 2.5 Data analysis

The data was analyzed by a one-way ANOVA within the General Linear Model (GLM) framework in SPSS 28.0 software (SPSS Inc., Chicago, IL), followed by Duncan’s multiple comparisons to investigate the differences between groups. All data were presented as means and standard error, and a *p*-values <0.05 was considered statistically significant.

## 3 Results

### 3.1 Growth performance and visceral index

As shown in Table 1, after two weeks of orally gavaging acetate gossypol, compared with the control group mice, the body weight of mice in AGC group, BP group and BT group reduced by 21.62, 17.84 and 13.64%, respectively (*p* < 0.05), and there was no significant difference in body weight among AGC group, BP group and BT group mice. In the first week of orally gavaging acetate gossypol, compared with the control group mice, the average daily feed intake of AGC group mice decreased (*p* < 0.05), in the second week of gavaging acetate gossypol, the average daily feed intake of AGC group mice was 47.25% lower than that of C group mice, compared with AGC group mice, the average daily feed intake of BP group and BT group increased by 40.47 and 45.69% (*p* < 0.05). The kidney index of mice in AGC group was lower than that in C group and BP group (*p* < 0.05), and the lung index of mice in AGC group, BP group and BT group was lower than that in the control group and BC group (*p* < 0.05).

**Table 1:** Effects of biotin on body weight, average daily feed intake, and visceral index in mice.

Project	C	BC	AGC	BP	BT	SEM	*p*-values
Body weight (g)							
0d	25.20	25.40	25.27	24.67	24.53	0.243	0.740
7d	31.87^ab^	32.00^ab^	33.20^a^	30.67^b^	30.53^b^	0.299	0.025
14d	38.33	38.33	37.93	38.07	38.07	0.382	0.997
21d	41.21^a^	42.59^a^	32.30^b^	33.86^b^	35.59^b^	0.709	<0.001
Average daily feed intake (g)							
1st wk	5.77	5.81	5.81	5.81	5.78	0.046	0.998
2nd wk	7.59^a^	7.54^a^	6.67^b^	7.14^ab^	7.48^a^	0.104	0.005
3rd wk	7.26^a^	7.37^a^	3.83^c^	5.38^b^	5.58^b^	0.237	<0.001
Visceral index							
Heart index	6.02^a^	5.21^b^	5.05^b^	5.11^b^	4.89^b^	0.089	<0.001
Liver index	45.85	46.05	46.83	47.34	48.21	0.448	0.452
Kidney index	16.06^a^	15.26^ab^	13.24^c^	14.63^b^	13.51^c^	0.208	<0.001
Spleen index	2.35	2.60	2.29	2.06	2.33	0.103	0.618
Lung index	6.61^a^	5.56^b^	5.10^b^	4.86^b^	5.27^b^	0.172	0.009

^a–cDemonstrate a statistically significant distinction, p < 0.05.^

### 3.2 Blood biochemistry

As shown in Table 2, that compared with the control group mice, the serum ALT, AST, TBIL, TP, LDL-C and BUN contents of AGC group mice (*p* < 0.05), and the contents of HDL-C and GLU were decreased (*p* < 0.05). Compared with the AGC group mice, the serum BUN content of BP group mice reduced by 15.38%, and the serum AST, TP and BUN contents of BT group mice reduced by 24.27, 7.39 and 10.73% (*p* < 0.05), respectively. Biotin had no significant effect on fluctuations of ALT, TBIL, HDL-C, LDL-C and GLU content in serum of acetate gossypol-treated mice.

**Table 2:** Effects of biotin on blood biochemical indices in mice.

Project	C	BC	AGC	BP	BT	SEM	*p*-values
ALT (U/L)	31.40^b^	34.10^b^	105.10^a^	89.60^a^	88.30^a^	7.766	0.001
AST (U/L)	101.20^c^	122.60^bc^	177.20^a^	157.30^ab^	134.20^bc^	6.338	<0.001
TBIL (μmol/L)	0.72^b^	0.75^b^	3.75^a^	3.43^a^	2.82^a^	0.266	<0.001
TP (g/L)	58.61^b^	57.09^b^	63.90^a^	62.12^a^	59.18^b^	0.552	<0.001
ALB (g/L)	21.61	25.04	27.62	28.20	26.96	0.960	0.185
ALP (U/L)	218.70	263.50	305.00	217.20	254.30	11.504	0.086
TG (mmol/L)	1.17	1.28	1.29	1.12	1.20	0.036	0.494
TC (mmol/L)	2.89	2.67	2.51	2.54	2.37	0.074	0.211
HDL-C (mmol/L)	2.52^a^	2.25^b^	1.58^c^	1.61^c^	1.55^c^	0.069	<0.001
LDL-C (mmol/L)	0.30^b^	0.24^b^	0.60^a^	0.60^a^	0.50^a^	0.036	<0.001
BUN (mmol/L)	6.96^c^	8.66^b^	9.69^a^	8.20^b^	8.65^b^	0.192	<0.001
GLU (mmol/L)	6.66^a^	5.61^ab^	2.90^c^	3.26^c^	4.57^bc^	0.359	0.002

^a–cDemonstrate a statistically significant distinction, p < 0.05. ALT, alanine aminotransferase; AST, aspartate aminotransferase; TBIL, total bilirubin; TP, total protein; ALB, albumin; ALP, alkaline phosphatase; TG, triglyceride; TC, total cholesterol; HDL-C, high density lipoprotein cholesterol; LDL-C, low density lipoprotein cholesterol; BUN, urea; GLU, glucose.^

### 3.3 Histopathological observation

As shown in Table 3, compared with the control group mice, the duodenum villus height of AGC group mice reduced by 19.94%, and the villus height/crypt depth of three intestinal segments of AGC group mice decreased significantly (*p* < 0.05). Biotin significantly increased the duodenum villus height and villus height/crypt depth in acetate gossypol-treated mice (*p* < 0.05). Biotin treatment significantly increased the jejunal villus height and the ratio of jejunal villus height to crypt depth of acetate gossypol-treated mice (*p* < 0.05). Compared with the control group mice, biotin reduced the jejunal crypt depth, and compared with the AGC group mice, biotin increased the jejunal villus height/crypt depth (*p* < 0.05).

**Table 3:** Effects of biotin on intestinal histomorphological in mice.

Project	C	BC	AGC	BP	BT	SEM	*p*-values
Duodenum							
Villus height (μm)	552.11^a^	539.97^a^	442.02^b^	582.20^a^	576.43^a^	13.830	0.005
Crypt depth (μm)	109.37^b^	112.69^ab^	124.79^ab^	129.93^a^	129.86^a^	2.732	0.032
Villus height/crypt depth	5.05^a^	4.85^ab^	3.57^c^	4.47^b^	4.50^ab^	0.106	<0.001
Jejunum							
Villus height (μm)	528.00^ab^	514.84^ab^	465.50^b^	540.69^a^	522.21^ab^	10.425	0.189
Crypt depth (μm)	105.41^b^	114.85^ab^	124.48^a^	126.09^a^	112.06^ab^	2.678	0.071
Villus height/crypt depth	5.07^a^	4.51^b^	3.77^c^	4.30^bc^	4.75^ab^	0.102	<0.001
Ileum							
Villus height (μm)	495.99^a^	448.99^ab^	444.4^ab^	376.76^c^	398.72^bc^	10.772	0.002
Crypt depth (μm)	120.78^ab^	112.93^bc^	132.02^a^	93.82^d^	101.23^cd^	2.790	<0.001
Villus height/crypt depth	4.09^a^	4.00^a^	3.38^b^	4.04^a^	3.94^a^	0.067	0.02

^a–dDemonstrate a statistically significant distinction, p < 0.05.^

As shown in Figure 1B, the arrows indicate the liver cells surrounding the vein. The sinusoids and portal triads of the control group and the BC group mice appear with no evidence of necrosis or inflammation. The liver cells of the AGC group were arranged in a disordered manner, the deposition of erythrocytes and plasma in the vein was obvious, indicating that acetate gossypol treatment caused certain liver damage in mice. The liver cells of the BP group and the BT group were arranged in a disordered manner, and there were blood cells between the liver cells. The liver cells in the BT group were arranged neatly, and there were blood cells between the liver cells, indicating that biotin treatment could alleviate the liver lesions caused by acetate gossypol to a certain extent.

## 4 Discussion

The continuous accumulation of low-dose gossypol in animals can cause poisoning, which is manifested as the decline of growth performance, damage of internal organs and reproductive performance. In this experiment, the body weight of mice was significantly reduced after one week of acetate gossypol administration, and the average daily feed intake of mice was significantly reduced after acetate gossypol administration. After two weeks of acetate gossypol administration, the feed intake of the AGC group mice was 47.25% lower than that of the control group. The body weight of grass carp treated with 400 mg/kg gossypol decreased significantly, the reason of this descent the intestinal physical barrier function was compromised (8). An experimental result indicated that dietary supplementation with gossypol at concentrations of 150 and 300 mg/kg in Nile tilapia significantly reduced the richness and diversity of the gut microbiota (9). Gossypol may exert inhibitory effects on animal growth performance, potentially by affecting intestinal metabolism.

Animal serum biochemical indicators can reflect the health of the body, and can understand whether animal glucose metabolism, lipid metabolism and liver function are normal. In this experiment, compared with the control group, the serum ALT, AST, TB, TP, ALP, LDL-C, and BUN content of mice fed with acetate gossypol were significantly increased, and the content of HDL-C and GLU was significantly decreased, indicating that acetate gossypol at a dose of 80 mg/(kg·BW) had an effect on glucose metabolism, lipid metabolism and liver and kidney function in mice. Compared with the control group, mice treated with acetate gossypol suspension at the dosage of 25 mg/kg body weight for two weeks have higher serum ALT content and lower HDL-C content (10). When the free gossypol content in the feed was 103.1 mg/kg, the daily weight gain of meat ducks was significantly reduced, and the serum ALT and AST activities of meat ducks increased linearly with the increase of the level of gossypol in the feed, with the increase of the content of gossypol in the diet and the prolongation of the intake time of gossypol, the damage degree of the liver of meat ducks was significantly deepened (11). Gossypol can cause damage to animal liver and we hope that biotin could reduce these damages.

In this study, compared with the control group, the content of BUN in the biotin control group was significantly increased, and the content of HDL-C was significantly decreased. Feeding 5-week-old male rats with diets containing 0.05, 0.10 and 0.20% biotin for three weeks, there was no significant difference in plasma ALT and AST levels among the experimental groups, indicating that the intake of biotin below 0.20% had no significant effect on the liver of mice (12). It was found that the serum GLU content of diabetic rats was significantly reduced after 12 weeks of treatment with 15 mg/kg gossypol, which was 64% lower than that of untreated diabetic rats, gossypol reduced serum glucose content by inhibiting liver glucose production through down-regulation of related genes, such as the messenger mRNA levels of phosphoenolpyruvate carboxykinase and glucose-6-phosphatase (13). Treated mice with oral glucose 1 g/kg and oral gossypol 1 mg/kg and 2.5 mg/kg at the same time, compared with the mice treated with oral glucose, gossypol treatment groups showed significantly reduced blood GLU levels, and 2.5 mg/kg gossypol treatment group had stronger ability to reduce blood GLU levels (14). Gossypol can reduce serum GLU levels in hyperglycemic animals, which is consistent with the trend of acetate gossypol in reducing the blood glucose content in mice in this experiment. The effect of biotin supplementation on animal islets have been studied and it was found that compared with the control group, the insulin secretion of islets in biotin-supplemented mice increased, the mRNA abundance of glucokinase, acetyl-CoA carboxylase and insulin increased, glucose tolerance increased, and glucose-stimulated serum insulin levels increased (15), indicating that biotin can be involved in the regulation of blood glucose.

Biotin may affect the content of HDL-C in serum of mice by regulating lipid metabolism. Biotin also affects the level of BUN in animal body. BUN is the main product of nitrogen metabolism, which is usually produced in the urea cycle of liver. Biotin may also indirectly affect the production of urea by affecting the islets of animals. Insulin not only promotes GLU uptake, but also promotes the synthesis and utilization of amino acids. When the level of insulin increases, the metabolism of amino acids accelerates, and the nitrogen produced also increases, eventually increasing the synthesis of urea (16). The study of Sawamura H et al. fed weaned rats with diets containing 0.01, 0.1 and 1.0% biotin for six weeks, no significant differences in serum TG, ALT, AST and BUN levels were observed among the groups. Intake of 1.0% and below biotin for six weeks did not lead to a significant increase in serum TG, ALT, AST and BUN levels, but the serum BUN content in the 1.0% biotin group was 21% higher than that in the control group (17), which was consistent with the trend of this test.

Through the experiments on rats and mice, it was found that biotin could reduce the increase of blood ammonia level caused by acute ammonia poisoning induced by ammonium acetate (18), indicating that after ammonia poisoning, biotin in animal blood could be rapidly detoxified, which was consistent with the fact that the BP group and the BT group could significantly reduce the increase of serum BUN content in acetate gossypol-treated mice. Biotin as a cofactor for biotin-dependent urea carboxylase, catalyzes urea decomposition into ammonia and carbon dioxide, facilitating nitrogen source utilization (19). Biotin has the effect of regulating BUN level in animal body. It shows that biotin can reduce the liver and kidney function damage caused by acetate gossypol to a certain extent, enhance the body’s resistance, and protect the liver and kidney of the body.

The histological morphology of animal intestine is closely related to the function of intestine. The ratio of intestinal epithelial villus height to crypt depth is directly related to the digestion and absorption ability of intestine, which is an important index to evaluate intestinal health and absorption ability. In this experiment, biotin significantly increased the duodenum villus height and villus height/crypt depth in acetate gossypol-treated mice, and biotin treatment significantly increased the jejunal ratio of jejunal villus height to crypt depth of acetate gossypol-treated mice. The villus height and the ratio of villus height to crypt depth in duodenum, jejunum and ileum of goslings treated with 50 mg/(kg·BW) gossypol were significantly decreased on day 7 and 14 (20). Studies have found that the intestinal barrier permeability of Nile tilapia fed a 300 mg/kg gossypol diet increased, the intestinal epithelial cells at the top of the intestinal villi fell off, and the villus height, villus width and basal layer thickness of the intestine were significantly reduced (21). Compared with the control group, when soybean meal was replaced by 10 and 20% cottonseed meal, the villus height and crypt depth of duodenum and jejunum were significantly reduced, and the ratio of villus height to crypt depth of duodenum and jejunum was also significantly reduced (22). Compared with the control group, the villus height and villus height/crypt depth of mice gavaged with 0.2 mL solution with free gossypol = 100.06 mg/kg every other day for ten weeks are decreased significantly, and crypt depth increased, this result has the same trend with our experiment (23). These results were consistent with the results of this experiment, indicating that gossypol damaged the intestinal morphology and digestion and absorption capacity of mice.

Biotin may mitigate acetate gossypol-induced hepatic and intestinal damage in mice by modulating immune factors and antioxidant capacity. Biotin deficiency impacts intestinal health, potentially exacerbating inflammation and contributing to inflammatory bowel disease pathogenesis (24, 25). Biotin-deficient mice exhibit elevated serum TNF-*α* levels compared to controls (26). *In vitro* studies demonstrate biotin’s inhibitory effect on IL-1β production (27), while cellular experiments reveal 4.3 and 5.6 fold increases in IFN-*γ* and IL-1β mRNA abundance with biotin supplementation (28). Biotin treatment reduces inflammatory cytokine expression, normalizing IL-6 levels in mice (29). Dietary biotin (1.62 mg/kg) restores hepatic/intestinal morphology and alleviates inflammation in turbot (30). Grass carp show minimal antioxidant enzyme activity and glutathione concentration at the lowest biotin dose (0.012 mg/kg) (31), whereas 0.33 mg/kg biotin significantly enhances superoxide dismutase activity in carp tissues and serum (32). These results suggest that biotin may have a certain effect on the integrity of intestinal morphology and function in animals. Combined with the results of our experiment, it was found that biotin can improve the intestinal and liver damage caused by acetate gossypol in mice.

## 5 Conclusion

In this study, the use of biotin potentially increased the average daily feed intake of acetate gossypol-treated mice, reduce the increase of serum AST, TP and BUN levels in acetate gossypol-treated mice, and increase the values of villus height/crypt depth in duodenum and jejunum in acetate gossypol-treated mice. Biotin has the potential to protect the morphological and functional integrity of the liver and intestine in acetate gossypol-treated mice. This experiment found that biotin has a certain protective effect on the liver and intestinal health of acetate gossypol-treated mice. In the future, we can continue to study the effect on growth performance, serum biochemical profiles, and morphology of acetate gossypol-treated mice.

## Data Availability

The original contributions presented in the study are included in the article/supplementary material, further inquiries can be directed to the corresponding author.
